# The association between composite inflammatory indicators and the clinicopathological characteristics of differentiated thyroid carcinoma

**DOI:** 10.3389/fmolb.2025.1660379

**Published:** 2025-09-02

**Authors:** Li-Yuan Yang, Li-Peng Yang

**Affiliations:** ^1^ Department of Medical Oncology, Brunch of Minhang, Fudan University Shanghai Cancer Hospital Shanghai, The People’s Republic of China, Shanghai, China; ^2^ Department of Pathology, Shanghai Sixth People’s Hospital Affiliated to Shanghai Jiao Tong University School of Medicine, Shanghai, China

**Keywords:** differentiated thyroid carcinoma, clinicopathological characteristics, monocyte-to-lymphocyte ratio, systemic inflammation response index, systemic immune-inflammation index

## Abstract

**Purpose:**

This study examined the associations between composite inflammatory indicators—including the Monocyte-to-Lymphocyte Ratio (MLR), Systemic Inflammation Response Index (SIRI), and Systemic Immune-Inflammation Index (SII)—and the clinicopathological characteristics in patients with Differentiated Thyroid Carcinoma (DTC). To provide a novel insight into refining patient selection criteria for active surveillance (AS) strategies in DTC patients.

**Methods:**

This retrospective study analyzed preoperative MLR, SIRI, and SII values in 231 DTC patients treated at Shanghai Sixth People’s Hospital Affiliated to Shanghai Jiao Tong University School of Medicine between January 2021 and February 2023. Comparisons of these inflammatory indicators were performed across subgroups stratified by clinicopathological characteristics. Subsequently, patients were categorized into low-expression and high-expression groups based on the median values of MLR, SIRI, and SII, followed by comparative analyses of clinicopathological features between the two groups.

**Results:**

Comparative Analysis: SIRI levels were significantly elevated (P < 0.05) in patients with larger maximum tumor diameter, higher Ki-67 index, lymph node metastasis (LNM), capsular invasion and bilateral thyroid tumors. Similarly, SII levels were significantly elevated (P < 0.05) in patients with larger maximum tumor diameter, aggressive pathologic variants, LNM, and capsular invasion. In contrast, the MLR showed no significant associations with any of the clinicopathological subgroups analyzed (all P > 0.05). Subgroup Analysis: Compared to the high-MLR group, the low-MLR group showed a significantly lower prevalence of psammoma bodies, and lower incidence of multifocal tumors (all P < 0.05). SIRI Group Comparisons: The high-SIRI group exhibited significantly larger maximum tumor diameter, higher rates of lymphovascular invasion compared to those in the low-SIRI group (P < 0.05). SII Group Comparisons: The high-SII group demonstrated a significantly higher prevalence of aggressive pathologic variants compared to the low-SII group (P < 0.05).

**Conclusion:**

Patients with DTC presenting elevated preoperative levels of MLR, SIRI, and SII demonstrated a significantly higher incidence of multiple adverse clinicopathological features postoperatively compared to those with lower baseline inflammatory indicators levels. These findings suggest that MLR, SIRI, and SII may serve as predictive biomarkers for adverse tumor clinicopathological characteristics in DTC.

## 1 Introduction

DTC encompassing papillary thyroid carcinoma (PTC), oncocytic follicular thyroid carcinoma (OFTC) and follicular thyroid carcinoma (FTC) as its subtypes, has demonstrated a progressively rising incidence in recent years, accompanied by a gradual trend toward younger age groups in disease onset (Li et al., 2020; Miranda-Filho et al., 2021). The clinicopathological characteristics of DTC have been shown to play a critical role in guiding therapeutic strategies and prognostic evaluation for patients. According to the American Thyroid Association (ATA) guidelines for thyroid cancer management, active surveillance (AS) is recommended as a viable alternative to immediate surgery for low-risk papillary thyroid carcinoma (PTC) with tumor diameters <1 cm. Furthermore, emerging studies from multiple research institutions have demonstrated promising outcomes in expanding the eligibility criteria for AS, with some prospective trials even extending the maximum tumor diameter threshold to ≤2 cm while maintaining oncologic safety (Ghai et al., 2024; Kim et al., 2024; Miyauchi et al., 2019; Miyauchi et al., 2023; Sawka et al., 2018).

Emerging evidence has demonstrated strong associations between inflammatory indicators and the clinicopathological features as well as prognosis of various solid malignancies. In cancers including but not limited to gastric, colorectal, ovarian, non-small cell lung cancer (NSCLC), and renal cell carcinoma, inflammation-related indicators such as the neutrophil-to-lymphocyte ratio (NLR), platelet-to-lymphocyte ratio (PLR), C-reactive protein (CRP), and prognostic nutritional index (PNI) have demonstrated significant predictive value for tumor aggressiveness and prognosis (Bagley et al., 2017; Luo et al., 2019; Meng et al., 2021; Numakura et al., 2024; Winata et al., 2024). However, research on the clinical relevance of these inflammation-related indicators in DTC remains limited, with existing studies yielding inconsistent conclusions regarding their correlations with clinicopathological characteristics and prognosis (Boi et al., 2017; Cai et al., 2024; Denk and Greten, 2022; Ferrari et al., 2019; Li et al., 2024; Pasquali et al., 2023).

The inflammatory indicators evaluated in this study were calculated using the following formulas: MLR: = M/L where M and L represent the absolute counts of peripheral blood monocytes and lymphocytes, respectively (Napolitano et al., 2022). SIRI:= N × M/L where N, M, and L represent the absolute counts of peripheral blood neutrophils, monocytes, and lymphocytes, respectively (Zuo et al., 2023). SII:=P × N/L where P, N, and L represent the absolute counts of peripheral platelets, neutrophils, and lymphocytes, respectively (Kou et al., 2023).

Previous studies have demonstrated that the MLR, SIRI, and SII are negatively correlated with prognosis in various solid tumors (Bagley et al., 2017; Luo, et al., 2019; Meng et al., 2021; Numakura et al., 2024; Winata et al., 2024). However, their associations with the clinicopathological characteristics of DTC patients remain underexplored. This study aims to investigate the relationships between preoperative MLR, SIRI, and SII and the clinicopathological characteristics of DTC patients, providing a novel insight into the potential application of these inflammatory indicators in guiding AS strategies for DTC. The findings are reported as follows.

## 2 Methods

### 2.1 Data collection

This study was conducted in strict adherence to the principles of the Declaration of Helsinki. Ethical approval was granted by the Ethics Committee of the Shanghai Sixth People’s Hospital Affiliated to Shanghai Jiao Tong University School of Medicine (Approval No. 2021-KY-109(K)). Given the retrospective nature of this study and the utilization of fully anonymized patient data, the requirement for written informed consent was formally waived. The final cohort comprised 231 patients.

Medical records of DTC patients treated at Shanghai Sixth People’s Hospital Affiliated to Shanghai Jiao Tong University School of Medicine between January 2021 and February 2023 were reviewed. Inclusion criteria: ① confirmed postoperative pathological diagnosis of DTC; ② availability of fasting blood samples collected between 8:00–10:00 a.m. preoperatively for complete blood count analysis. Exclusion criteria: ① concurrent conditions affecting white blood cell counts; ② history of other malignancies; ③ preoperative use of medications affecting inflammatory cells. Based on the above criteria, 231 eligible patients were ultimately enrolled in this study.

Collection of Baseline Clinical Data: Clinical documentation must include baseline characteristics (age, gender, body mass index), hematologic indices such as complete blood count (CBC).

Collection of pathological data: The histopathological diagnostic report include assessment of maximum tumor diameter, histological subtype, lymphovascular invasion status, presence of psammoma bodies, tumor-infiltrating lymphocytes (TILs), concurrent Hashimoto’s thyroiditis (HT), thyroid capsular invasion, multifocality (defined as ≥2 tumor foci in the surgical specimen), bilateral thyroid tumors, LNM, genetic mutation profile, and Ki-67 index.

Evaluate the differences in MLR, SIRI, and SII values among patients with distinct clinicopathological characteristics, including maximum tumor diameter, LNM, and lymphovascular invasion, the patient cohort was stratified into the following groups based on the median values of these indicators: low-MLR group (MLR < median), high-MLR group (MLR ≥ median), low-SIRI group (SIRI < median), high-SIRI group (SIRI ≥ median), low-SII group (SII < median), and high-SII group (SII ≥ median). The differential distribution of clinicopathological characteristics (e.g., maximum tumor diameter, LNM, and lymphovascular invasion) across these groups was subsequently analyzed.

### 2.2 Statistical analysis

Data analysis was performed using R statistical software (version 4.4.1). Categorical variables were presented as frequencies and percentages (%), with intergroup comparisons conducted using the chi-square test. Continuous variables were expressed as mean ± standard deviation (SD) and analyzed with the Student’s t-test for group comparisons. Comparisons among multiple groups were assessed using analysis of variance (ANOVA). P-value<0.05 was considered statistically significant. Fisher’s exact test governed by sample size and expected frequency thresholds was supplemented by residual decomposition analysis to isolate significant contributors. Separate univariable and multivariable logistic regression models were performed treating aggressive pathological variants and lymph node metastasis as distinct dependent variables. ROC analysis was used to evaluate the predictive performance of inflammatory markers for lymph node metastasis and their ability to discriminate aggressive pathologic variants.

## 3 Results

### 3.1 Clinical Characteristics

This study included 231 patients [78 males (33.8%) and 153 females (66.2%) ]with an age range of 17–80 years (mean: 43.71 ± 12.9 years). Body mass index (BMI) ranged from 16.26 to 34.29 kg/m^2^ (mean: 24.04 ± 3.51 kg/m^2^), and maximum tumor diameter varied between 0.1 and 6.5 cm (mean: 1.16 ± 1.06 cm). The MLR ranged from 0.08 to 1.33 (mean: 0.28 ± 0.16; median: 0.24). The SIRI ranged from 0.25 to 13.2 (mean: 1.40 ± 1.70; median: 0.94), while the SII ranged from 148.89 to 6,509 (mean: 725.94 ± 724.69; median: 520.59). Patients were dichotomized into high and low groups for MLR, SIRI, and SII using the median value of each index within the study cohort.

### 3.2 Differences in preoperative MLR, SIRI, and SII values among DTC patients with different clinicopathological characteristics

#### 3.2.1 SIRI and MLR

SIRI levels differed significantly across the following clinicopathological subgroups (all P < 0.05): tumors >1 cm vs. ≤ 1 cm in maximum tumor diameter, high KI67 index (≥3%) vs. low KI67 (<3%), LNM-positive vs. LNM-negative, capsular invasion vs. non-invasion, and bilateral vs. unilateral thyroid tumors. Specifically, patients with larger maximum tumor diameter (>1 cm) exhibited higher SIRI levels compared to those with smaller maximum tumor diameter (≤1 cm). Similarly, elevated SIRI levels were observed in subgroups with high KI67 index (≥3%), LNM-positive, capsular invasion, and bilateral thyroid tumors (Table 1). In contrast, no statistically significant differences in MLR levels were observed among the subgroups (Table 2).

**TABLE 1:** SIRI levels in DTC patients with different clinicopathological characteristics (n = 231).

Clinicopathological characteristic	Number of cases	SIRI (mean ± SD)	t-value	P-value
Gender			1.7071	0.0904
Male	78	1.69 ± 2.06		
Female	153	1.25 ± 1.47		
Age (years)			0.086251	0.9314
<55	179	1.40 ± 1.80		
≥55	52	1.38 ± 1.33		
BMI (kg/m^2^)			−0.3453	0.7308
Normal (18.5 ≤ BMI <25)	153	1.41 ± 1.56		
Overweight (25 ≤ BMI <30)	58	1.52 ± 2.25		
Maximum Tumor Diameter (cm)			−2.2294	0.0280
0–1	152	1.18 ± 1.24		
>1	79	1.81 ± 2.31		
Aggressive Pathologic Variants			−1.9741	0.0527
Yes	57	1.94 ± 2.67		
No	174	1.21 ± 1.19		
Pathologic Variants				
Classic	162	1.23 ± 1.22		
Tall cell variant	36	1.73 ± 2.21	−1.29	0.20
Diffuse sclerosing variant	3	1.28 ± 0.66	/	/
Hobnail variant	15	1.96 ± 2.62	−1.05	0.31
Solid variant	3	4.84 ± 7.24	/	/
Follicular variant	9	0.88 ± 0.65	/	/
FTC	3	1.26 ± 0.32	/	/
Lymphovascular Invasion			−1.3917	0.1682
Yes	58	1.73 ± 2.28		
No	173	1.29 ± 1.45		
Psammoma Bodies			0.21219	0.8325
Yes	34	1.36 ± 0.93		
No	197	1.40 ± 1.80		
Ki-67 Index (%)			−2.0241	0.0442
<3	101	1.16 ± 1.22		
≥3	130	1.58 ± 1.98		
Genetic Mutation Status				
Wild-type	18	1.40 ± 1.70		
BRAF mutation	186	1.19 ± 1.00	1.5307	0.1267
Non-BRAF mutation	27	2.78 ± 3.61	−1.959	0.0604
Tumor-Infiltrating Lymphocytes			0.95108	0.3430
Yes	64	1.25 ± 1.32		
No	167	1.45 ± 1.83		
Concurrent Hashimoto’s Thyroiditis			−0.38868	0.7
Yes	28	1.52 ± 1.83		
No	203	1.38 ± 1.69		
Lymph Node Metastasis			−2.3184	0.0219
Yes	106	1.69 ± 2.27		
No	125	1.15 ± 0.94		
Capsular Invasion			−2.5141	0.0140
Yes	69	1.96 ± 2.57		
No	162	1.16 ± 1.07		
Multifocal Tumor			0.031124	0.9752
Yes	55	1.39 ± 1.56		
No	176	1.40 ± 1.75		
Bilateral Thyroid Tumor			−2.1977	0.0325
Yes	47	2.11 ± 2.71		
No	184	1.22 ± 1.28		

**TABLE 2:** MLR levels in DTC patients stratified by clinicopathological characteristics (n = 231).

Clinicopathological characteristic	Number of cases	MLR (mean ± SD)	t-value	P-value
Gender			1.4199	0.1584
Male	78	0.3 ± 0.2		
Female	153	0.27 ± 0.13		
Age (years)			−0.25329	0.8006
<55	52	0.28 ± 0.16		
≥55	179	0.28 ± 0.14		
BMI(kg/m^2^)			1.1567	0.2503
Underweight (<18.5)	4	0.29 ± 0.12		
Normal (18.5 ≤ BMI <25)	153	0.29 ± 0.16		
Overweight (25 ≤ BMI <30)	58	0.26 ± 0.18		
Obese (BMI ≥30)	16	0.22 ± 0.04		
Maximum Tumor Diameter (cm)			−1.6213	0.1078
0–1	130	0.26 ± 0.13		
>1	101	0.30 ± 0.19		
Aggressive Pathologic Variants			−1.4611	0.1488
Yes	57	0.32 ± 0.24		
No	174	0.27 ± 0.12		
Pathologic Variants				
Classic	162	0.27 ± 0.12		
Tall cell variant	36	0.28 ± 0.19	−0.54	0.59
Diffuse sclerosing variant	3	0.27 ± 0.05	/	/
Hobnail variant	15	0.36 ± 0.31	−1.13	0.28
Solid variant	3	0.49 ± 0.44	/	/
Follicular variant	9	0.26 ± 0.12	/	/
FTC	3	0.28 ± 0.15	/	/
Lymphovascular Invasion			−1.455	0.1499
Yes	58	0.31 ± 0.21		
No	173	0.27 ± 0.14		
Psammoma Bodies			−0.26345	0.7930
Yes	34	0.28 ± 0.10		
No	197	0.28 ± 0.17		
Ki-67 Index (%)			−1.9277	0.0551
<3	101	0.26 ± 0.13		
≥3	130	0.30 ± 0.17		
Genetic Mutation Status				
Wild-type	18	0.30 ± 0.20		
BRAF mutation	186	0.26 ± 0.11	0.52954	0.6007
Non-BRAF mutation	27	0.38 ± 0.30	−0.41369	0.6813
Tumor-Infiltrating Lymphocytes			0.025808	0.9795
Yes	64	0.28 ± 0.15		
No	167	0.28 ± 0.16		
Concurrent Hashimoto’s Thyroiditis			−0.58598	0.5620
Yes	28	0.30 ± 0.19		
No	203	0.28 ± 0.15		
Lymph Node Metastasis			−1.7846	0.0762
Yes	106	0.30 ± 0.20		
No	125	0.26 ± 0.11		
Capsular Invasion			−1.8194	0.0723
Yes	69	0.31 ± 0.22		
No	162	0.26 ± 0.12		
Multifocal Tumor			−1.012	0.3146
Yes	55	0.30 ± 0.18		
No	176	0.27 ± 0.15		
Bilateral Thyroid Tumor			−1.8245	0.0738
Yes		0.33 ± 0.24		
No		0.27 ± 0.12		

#### 3.2.2 SII

SII levels showed statistically significant differences among the following groups (all P < 0.05): tumors >1 cm vs. ≤1 cm in maximum diameter, aggressive pathologic variants vs. non-aggressive pathologic variants, LNM-positive vs. LNM-negative, and capsular invasion vs. non-invasion. Patients with larger maximum tumor diameter (>1 cm), aggressive pathologic variants, LNM-positive, and capsular invasion demonstrated significantly higher SII levels compared to their respective counterparts (Table 3).

**TABLE 3:** SII levels in DTC patients with different clinicopathological characteristics (n = 231).

Clinicopathological feature	Number of cases	SII (x̄ ± SD)	t-value	P-value
Gender			0.83655	0.4045
Male	78	788.15 ± 883.89		
Female	153	694.23 ± 629.21		
Age (years)			0.16597	0.8685
<55	52	729.67 ± 760.23		
≥55	179	713.09 ± 592.51		
BMI (kg/m^2^)			−0.76713	0.4456
Normal (18.5 ≤ BMI <25)	153	705.51 ± 570.30		
Overweight (25 ≤ BMI <30)	58	821.92 ± 1,101.05		
Maximum Tumor Diameter (cm)			−3.2343	0.0017
0–1	152	585.59 ± 340.62		
>1	79	995.99 ± 1,100.79		
Aggressive Pathologic Variants			−2.3816	0.0203
Yes	57	1,015.30 ± 1,190.19		
No	174	631.15 ± 450.61		
Pathologic Variants				
Classic	162	640.70 ± 461.20		
Tall cell variant	36	888.07 ± 1,114.98	−1.31	0.20
Diffuse sclerosing variant	3	1,161.4 ± 1,278.27	/	/
Hobnail variant	15	1,018.50 ± 851.90	/	/
Solid variant	3	2,168.45 ± 3,044.19	/	/
Follicular variant	9	576.56 ± 226.26	/	/
FTC	3	491.00 ± 195.45	/	/
Lymphovascular Invasion			−1.4517	0.1508
Yes	58	870.53 ± 944.81		
No	173	677.47 ± 630.11		
Psammoma Bodies			−0.52177	0.6037
Yes	34	772.10 ± 517.01		
No	197	717.97 ± 755.54		
Ki-67 Index (%)			−1.5504	0.1225
<3	101	647.27 ± 502.16		
≥3	130	787.06 ± 855.73		
Genetic Mutation Status				
Wild-type	18	685.07 ± 611.97		
BRAF mutation	186	655.86 ± 593.63	0.19384	0.8482
Non-BRAF mutation	27	1,235.97 ± 1,259.77	−1.9528	0.0579
Tumor-Infiltrating Lymphocytes			0.97184	0.3324
Yes	64	665.74 ± 473.64		
No	167	749.01 ± 800.36		
Concurrent Hashimoto’s Thyroiditis			0.15496	0.8775
Yes	28	711.24 ± 498.26		
No	203	727.97 ± 751.50		
Lymph Node Metastasis			−1.994	0.04801
Yes	106	833.80 ± 939.17		
No	125	634.48 ± 457.00		
Capsular Invasion			−2.6154	0.0105
Yes	69	953.36 ± 959.28		
No	162	629.08 ± 574.44		
Multifocal Tumor			0.5292	0.5976
Yes	55	688.62 ± 531.91		
No	176	737.60 ± 776.11		
Bilateral Thyroid Tumor			−1.3824	0.1713
Yes	47	859.26 ± 746.78		
No	184	691.89 ± 717.04		

### 3.3 Comparative analysis of clinicopathological characteristics between low and high MLR, SIRI, and SII groups

#### 3.3.1 Comparative analysis of clinicopathological characteristics between low and high MLR groups

Comparison between the low MLR group and the high MLR group revealed no statistically significant differences (p > 0.05) in gender, age, maximum tumor diameter, proportion of aggressive pathologic variants, presence of lymphovascular invasion, Ki67 index, gene mutation profile, presence of TILs, concurrent Hashimoto’s thyroiditis, LNM status, capsular invasion status, and presence of bilateral thyroid tumors. However, among patients younger than 55 years, those in the low MLR group were significantly younger than those in the high MLR group (P < 0.05). Additionally, the low MLR group exhibited significantly higher BMI (P < 0.05), a significantly lower proportion of psammoma bodies (P < 0.05), and a significantly lower proportion of multifocal tumors (P < 0.05) compared to the high MLR group (Table 4).

**TABLE 4:** Comparison of clinicopathological characteristics between low and high MLR groups (n = 231).

Clinical feature	Low MLR group (n = 115)		High MLR group (n = 116)	χ^2^/t-value	P-value
Gender [n (%)]				0.0084917	0.9266
Male	38 (33.04%)		40 (34.48%)		
Female	77 (66.96%)		76 (65.52%)		
Age (years, mean ± SD)	42.29 ± 13.52		45.12 ± 12.16	−1.6741	0.0955
<55	36.63 ± 8.43		40.12 ± 8.76	−2.7156	0.0073
≥55	62.64 ± 7.32		61.59 ± 5.23	0.58967	0.5585
BMI (kg/m^2^, mean ± SD)	24.57 ± 3.77		23.51 ± 3.15	2.3165	0.0215
Normal (18.5 ≤ BMI <25)	22.16 ± 1.68		22.23 ± 1.55	−0.27156	0.7864
Overweight (25 ≤ BMI <30)	27.11 ± 1.37		27.19 ± 1.51	−0.18965	0.8504
Maximum Tumor Diameter (cm, mean ± SD)	1.07 ± 0.98		1.25 ± 1.12	−1.3399	0.1816
0–1	0.62 ± 0.21		0.64 ± 0.22	−0.5781	0.5641
>1	1.98 ± 1.27		1.98 ± 1.25	−0.01995	0.9841
Lymphovascular Invasion			3.6545	0.0559
Yes	23 (20.00%)		37 (31.90%)		
No	92 (80.00%)		79 (68.10%)		
Psammoma Bodies				4.0622	0.0439
Yes	11 (9.56%)		23 (19.23%)		
No	104 (90.43%)		93 (80.17%)		
KI67 Index (%)	2.70 ± 1.41		3.23 ± 3.77	−1.4118	0.1601
<3	1.55 ± 0.54		1.60 ± 0.49	−0.55415	0.5807
≥3	3.69 ± 1.14		4.38 ± 4.59	−1.1983	0.2345
Gene Mutation Status					
Wild-type	9 (7.83%)		9 (7.76%)		
BRAF mutation	95 (82.61%)		91 (78.44%)	2.8059 × 10^30^	1
Non-BRAF mutation	11 (9.57%)		16 (13.79%)	0.09375	0.7595
Tumor-Infiltrating Lymphocytes			0.48211	0.4875
Yes	29 (25.22%)		35 (30.17%)		
No	86 (74.78%)		81 (69.83%)		
Concurrent Hashimoto’s Thyroiditis			0.031386	0.8594
Yes	13 (11.30%)		15 (12.93%)		
No	102 (88.70%)		101 (87.07%)		
Lymph Node Metastasis			0.11258	0.7372
Yes	51 (44.35%)		55 (47.41%)		
No	64 (55.65%)		61 (52.59%)		
Capsular Invasion			1.945	0.1631
Yes	29 (25.22%)		40 (34.48%)		
No	86 (74.78%)		76 (65.52%)		
Multifocal Tumor				7.5288	0.0061
Yes	18 (15.65%)		37 (31.90%)		
No	97 (84.35%)		79 (68.10%)		
Bilateral Thyroid Tumor			0.89751	0.3434
Yes	20 (17.39%)		27 (23.28%)		
No	95 (82.61%)		89 (76.72%)		
Aggressive Pathologic Variants			0.0014181	0.9700
Yes	29 (25.22%)		28 (24.14%)		
No	86 (74.78%)		88 (75.86%)		
Pathologic Variants	n (%)	Residual	n (%)	Residual	0.182
Classic	80 (69.57%)	−0.12	82 (70.69%)	+0.12	
Tall cell variant	21 (18.26%)	+1.12	15 (12.93%)	−1.12	
Diffuse sclerosing variant	0 (0%)	−1.22	3 (2.59%)	+1.22	
Hobnail variant	7 (6.09%)	−0.07	8 (6.90%)	+0.07	
Solid variant	1 (0.87%)	−0.40	2 (1.72%)	+0.40	
Follicular variant	5 (4.35%)	+0.45	4 (3.45%)	−0.45	
FTC	1 (0.87%)	−0.40	2 (1.72%)	+0.40	

#### 3.3.2 Comparative analysis of clinicopathological characteristics between low and high SIRI groups

No significant associations were observed between SIRI groups (low SIRI group vs. high SIRI group) and the following parameters: gender, age, BMI, proportion of aggressive pathologic variants, presence of psammoma bodies, Ki67 index, gene mutation status, TILs, prevalence of concurrent Hashimoto’s thyroiditis, LNM, capsular invasion, multifocal tumor, and bilateral thyroid tumor (all P > 0.05). However, patients in the high SIRI group exhibited significantly larger maximum tumor diameter and a significantly higher incidence of lymphovascular invasion (both P < 0.05) (Table 5).

**TABLE 5:** Comparative analysis of clinicopathological characteristics between low/high SIRI groups (n = 231).

Clinicopathological characteristics	Low SIRI group (n = 117)		High SIRI group (n = 114)	χ2/t-value	P-value
Gender [n (%)]				1.941	0.1636
Male	34 (29.06%)		44 (38.60%)		
Female	83 (70.94%)		70 (61.40%)		
Age (years, mean ± SD)	43.03 ± 12.99		44.40 ± 12.84	−0.80562	0.4213
<55	37.47 ± 8.53		39.28 ± 8.92	−1.3905	0.1661
≥55	61.59 ± 6.23		62.64 ± 6.42	−0.59653	0.5535
BMI(kg/m2, mean ± SD)	24.13 ± 3.74		23.94 ± 3.26	0.41656	0.6774
Normal (18.5 ≤ BMI <25)	22.09 ± 1.68		22.29 ± 1.53	−0.78878	0.4315
Overweight (25 ≤ BMI <30)	27.22 ± 1.27		27.03 ± 1.61	0.48619	0.6292
Maximum Tumor Diameter (cm, mean ± SD)	0.94 ± 0.73		1.39 ± 1.27	−3.217	0.0015
0–1	0.62 ± 0.21		0.65 ± 0.23	−0.78332	0.4348
>1	1.75 ± 0.91		2.4 ± 1.41	−2.5398	0.0130
Lymphovascular Invasion			4.3553	0.0369
Yes	22 (18.80%)		36 (31.58%)		
No	95 (81.20%)		78 (68.42%)		
Psammoma Bodies				0.40855	0.5227
Yes	15 (12.82%)		19 (16.67%)		
No	102 (87.18%)		95 (83.33%)		
KI67 Index (%)	2.68 ± 1.51		3.27 ± 3.76	−1.576	0.1172
<3	1.51 ± 0.54		1.65 ± 0.48	−1.4069	0.1626
≥3	3.71 ± 1.32		4.37 ± 4.54	−1.142	0.2569
Gene Mutation Status			1.8622	0.3941
Wild-type	11 (9.40%)		7 (6.14%)		
BRAF mutation	95 (81.20%)		91 (79.82%)		
Non-BRAF mutation	11 (9.40%)		16 (14.04%)		
Tumor-Infiltrating Lymphocytes			2.2353	0.1349
Yes	38 (32.48%)		26 (22.81%)		
No	79 (67.52%)		88 (77.19%)		
Concurrent Hashimoto’s Thyroiditis				0.01646	0.8979
Yes	15 (12.82%)		13 (11.40%)		
No	102 (87.18%)		101 (88.60%)		
Lymph Node Metastasis			0.334	0.5633
Yes	51 (43.59%)		55 (48.25%)		
No	66 (56.41%)		59 (51.75%)		
Capsular Invasion				2.4539	0.1172
Yes	29 (24.79%)		40 (35.09%)		
No	88 (75.21%)		74 (64.91%)		
Multifocal Tumor				0.17584	0.6750
Yes	26 (22.22%)		29 (25.44%)		
No	91 (77.78%)		85 (74.56%)		
Bilateral Thyroid Tumor			1.1674	0.2799
Yes	20 (17.09%)		27 (23.68%)		
No	97 (82.91%)		87 (76.32%)		
Aggressive Pathologic Variants			1.0583	0.3036
Yes	25 (21.37%)		32 (28.07%)		
No	92 (78.63%)		82 (71.93%)		
Pathologic Variants	n (%)	Residual	n (%)	Residual	0.233
Classic	84 (73.04%)	+0.37	78 (67.24%)	−0.37	
Tall cell variant	19 (16.52%)	+0.25	17 (14.66%)	−0.25	
Diffuse sclerosing variant	1 (0.87%)	−0.40	2 (1.72%)	+0.40	
Hobnail variant	4 (3.48%)	−1.27	11 (3.45%)	+1.26	
Solid variant	1 (0.87%)	−0.40	2 (1.72%)	+0.40	
Follicular variant	6 (5.22%)	+0.72	3 (2.59%)	−0.71	
FTC	0 (0%)	−1.22	3 (2.59%)	+1.22	

#### 3.3.3 Comparative analysis of clinicopathological characteristics between low and high SII groups

No significant associations were observed between SII groups (low SII group vs. high SII group) and the following parameters: gender, BMI, maximum tumor diameter, lymphovascular invasion, presence of psammoma bodies, Ki67 index, gene mutation status TILs, prevalence of concurrent Hashimoto’s thyroiditis, LNM, capsular invasion, multifocal tumor, and bilateral thyroid tumor (all P > 0.05). Notably, among patients aged <55 years, those in the high SII group were significantly older than those in the low SII group (P < 0.05). Additionally, the high SII group had a significantly higher proportion of aggressive pathologic variants compared to the low SII group (P < 0.05). Fisher’s exact test revealed no statistically significant difference in the distribution of pathologic variants between the low and high systemic immune-inflammation index groups (P = 0.215). However, standardized residual analysis identified clinically meaningful patterns: The classic variant was significantly more frequent than expected in the low-SII group (residual +1.90, P = 0.057) while substantially less frequent in the high-SII group (residual −1.90). The hobnail variant demonstrated a non-significant enrichment trend in the high-SII group (residual +1.24, P = 0.215) (Table 6).

**TABLE 6:** Comparative analysis of clinicopathological characteristics between low- and high-SII groups (n = 231).

Clinicopathological characteristics	Low-SII group (n = 116)	High-SII group (n = 115)		χ2/t-value	P-value
Gender [n (%)]				0.1372	0.7111
Male	41 (35.34%)	37 (32.17%)			
Female	75 (64.66%)	78 (67.83%)			
Age (years, mean ± SD)	42.98 ± 13.51	44.44 ± 12.29		−0.85985	0.3908
<55	36.77 ± 8.81	39.88 ± 8.46		−2.4065	0.0171
≥55	61.62 ± 5.60	62.70 ± 7.13		−0.59255	0.5567
BMI (kg/m2, mean ± SD)	23.67 ± 3.55	24.40 ± 3.44		−1.5824	0.1149
Normal (18.5 ≤ BMI <25)	22.01 ± 1.61	22.40 ± 1.58		−1.4944	0.1372
Overweight (25 ≤ BMI <30)	27.06 ± 1.42	27.22 ± 1.43		−0.4163	0.6788
Maximum Tumor Diameter (cm, mean ± SD)	1.04 ± 0.93	1.28 ± 1.16		−1.7372	0.0838
0–1	0.63 ± 0.20	0.62 ± 0.24		0.097976	0.9221
>1	2.07 ± 1.23	2.26 ± 1.29		−0.66602	0.5076
Lymphovascular Invasion				0.036034	0.8494
Yes	28 (24.14%)	30 (26.09%)			
No	88 (75.86%)	85 (73.91%)			
Psammoma Bodies				0.91372	0.3391
Yes	14 (12.07%)	20 (17.39%)			
No	102 (87.93%)	95 (82.61%)			
KI67 Index (%)	2.74 ± 1.58	3.2 ± 3.72		−1.2164	0.2257
<3	1.51 ± 0.50	1.64 ± 0.53		−1.3274	0.1875
≥3	3.78 ± 1.42	4.31 ± 4.56		−0.91611	0.3624
Gene Mutation Status				1.2299	0.5407
Wild-type	10 (8.62%)	8 (6.96%)			
BRAF mutation	95 (81.90%)	91 (79.13%)			
Non-BRAF mutation	11 (9.48%)	16 (13.91%)			
Tumor-Infiltrating Lymphocytes				1.2093 × 10^−30^	1
Yes	32 (27.59%)	32 (27.83%)			
No	84 (72.41%)	83 (72.17%)			
Concurrent Hashimoto’s Thyroiditis				6.2177 × 10^−32^	1
Yes	14 (12.07%)	14 (12.17%)			
No	102 (87.93%)	101 (87.83%)			
Lymph Node Metastasis				0.51953	0.4710
Yes	50 (43.10%)	56 (48.70%)			
No	66 (56.90%)	59 (51.30%)			
Capsular Invasion				0.38188	0.5366
Yes	32 (27.59%)	37 (32.17%)			
No	84 (72.41%)	78 (67.83%)			
Multifocal Tumor				1.4377	0.2305
Yes	32 (27.59%)	23 (20.00%)			
No	84 (72.41%)	92 (80.00%)			
Bilateral Thyroid Tumor				0.47197	0.4921
Yes	21 (18.10%)	26 (22.61%)			
No	95 (81.90%)	89 (77.39%)			
Aggressive Pathologic Variants				4.7275	0.0297
Yes	21 (18.10%)	36 (31.30%)			
No	95 (81.90%)	79 (68.70%)			
Pathologic Variants	n (%)	Residual	n (%)	Residual	0.215
Classic	87 (75.65%)	+1.90	75 (64.66%)	−1.90	
Tall cell variant	16 (13.91%)	−0.82	20 (17.24%)	+0.82	
Diffuse sclerosing variant	1 (0.87%)	−0.40	2 (1.72%)	+0.40	
Hobnail variant	5 (4.35%)	−1.25	10 (8.62%)	+1.24	
Solid variant	1 (0.87%)	−0.40	2 (1.72%)	+0.40	
Follicular variant	3 (2.61%)	−0.92	6 (5.17%)	+0.91	
FTC	2 (1.74%)	+0.80	1 (0.86%)	−0.79	

#### 3.3.4 Univariate and multivariate analyses with aggressive pathological variants and lymph node metastasis as dependent variables

Univariate and multivariate analyses with aggressive pathological variants as the dependent variable revealed that Lymphovascular Invasion ( B=1.176,P=0.010 ), Age ( B=‐0.038,P=0.011 ), Ki-67 ( B=0.342,P=0.003 ), and SII ( B=0.434,P=0.030 ) remained significantly associated with aggressive variants after adjustment (p < 0.05 for all). This identifies Lymphovascular Invasion, Age, Ki-67, and SII as independent predictors of aggressive pathological variants (Table 7; Table 8).

**TABLE 7:** Univariate analyses with aggressive pathological variants as the dependent variable.

Clinicopathological characteristics	Aggressive		$\chi^{2}/Z$	P
	No	Yes		
Gender			3.447	0.063
Female	121 (79.08%)	32 (20.92%)		
Male	53 (67.95%)	25 (32.05%)		
Capsular Invasion			2.751	0.097
NO	127 (78.40%)	35 (21.60%)		
YES	47 (68.12%)	22 (31.88%)		
Multifocality			0.262	0.609
NO	134 (76.14%)	42 (23.86%)		
YES	40 (72.73%)	15 (27.27%)		
Bilaterality			4.195	0.041
NO	144 (78.26%)	40 (21.74%)		
YES	30 (63.83%)	17 (36.17%)		
Lymphovascular Invasion			19.941	<0.001
NO	143 (82.66%)	30 (17.34%)		
YES	31 (53.45%)	27 (46.55%)		
Psammoma Bodies			5.841	0.016
NO	154 (78.17%)	43 (21.83%)		
YES	20 (58.82%)	14 (41.18%)		
Non-BRAF mutation			0.027	0.869
NO	157 (75.48%)	51 (24.52%)		
YES	17 (73.91%)	6 (26.09%)		
BRAF mutation			0.124	0.724
NO	30 (73.17%)	11 (26.83%)		
YES	144 (75.79%)	46 (24.21%)		
BRAF and TERT mutation			0.732	0.392
NO	166 (76.15%)	52 (23.85%)		
YES	8 (61.54%)	5 (38.46%)		
RAS and TERT mutation			——	1.000
NO	173 (75.22%)	57 (24.78%)		
YES	1 (100.00%)	0 (0.00%)		
Tumor-Infiltrating Lymphocytes			2.059	0.151
NO	130 (77.84%)	37 (22.16%)		
YES	44 (68.75%)	20 (31.25%)		
Concurrent Hashimoto’s Thyroiditis			0.260	0.610
NO	154 (75.86%)	49 (24.14%)		
YES	20 (71.43%)	8 (28.57%)		
Age	43.00 (21.00)	36.00 (19.50)	−2.416	0.016
BMI	23.09 (4.49)	23.84 (5.17)	−1.567	0.117
Tumor Size	0.70 (0.63)	1.20 (1.10)	−5.256	<0.001
KI67	3.00 (1.00)	3.00 (3.00)	−3.219	0.001
MLR	0.24 (0.13)	0.23 (0.16)	−0.258	0.796
SIRI	0.91 (0.88)	1.03 (0.81)	−1.102	0.270
SII	491.67 (356.71)	572.14 (593.22)	−2.586	0.010

**TABLE 8:** Multivariate analyses with aggressive pathological variants as the dependent variable.

Variables	B	SE	Wald	P
Bilaterality	0.316	0.417	0.574	0.449
Lymphovascular Invasion	1.176	0.456	6.656	0.010
Psammoma Bodies	−0.316	0.520	0.370	0.543
Age	−0.038	0.015	6.391	0.011
Tumor Size	0.117	0.183	0.414	0.520
KI67	0.342	0.117	8.605	0.003
SII	0.434	0.2	4.709	0.030

Univariate and multivariate analyses with lymph node metastasis as the dependent variable demonstrated that Gender ( B=0.840,P=0.011 ), Psammoma Bodies ( B=1.144,P=0.040 ), Non-BRAF mutation ( B=‐1.544,P=0.021 ), Tumor Size ( B=0.456,P=0.047 ), and SIRI ( B=0.380,P=0.011 ) were significantly associated with lymph node metastasis (p < 0.05 for all). This identifies Gender, Psammoma Bodies, Non-BRAF mutation, Tumor Size, and SIRI as independent predictors of lymph node metastasis (Table 9; Table 10).

**TABLE 9:** Univariate analyses with Lymph node metastasis as the dependent variable.

Clinicopathological characteristics	Lymph node metastasis		$\chi^{2}/Z$	P
	No	Yes		
Gender			11.617	<0.001
Female	95 (62.09%)	58 (37.91%)		
Male	30 (38.46%)	48 (61.54%)		
Capsular Invasion			10.698	<0.001
NO	99 (61.11%)	63 (38.89%)		
YES	26 (37.68%)	43 (62.32%)		
Multifocality			9.158	0.002
NO	105 (59.66%)	71 (40.34%)		
YES	20 (36.36%)	35 (63.64%)		
Bilaterality			7.650	0.006
NO	108 (58.70%)	76 (41.30%)		
YES	17 (36.17%)	30 (63.83%)		
Pathologic Variants			0.125	0.723
Classic	93 (53.45%)	81 (46.55%)		
Aggressive Variants	32 (56.14%)	25 (43.86%)		
Lymphovascular Invasion			21.946	<0.001
NO	109 (63.01%)	64 (36.99%)		
YES	16 (27.59%)	42 (72.41%)		
Psammoma Bodies			12.268	<0.001
NO	116 (58.88%)	81 (41.12%)		
YES	9 (26.47%)	25 (73.53%)		
Non-BRAF mutation			4.033	0.045
NO	108 (51.92%)	100 (48.08%)		
YES	17 (73.91%)	6 (26.09%)		
BRAF mutation			0.571	0.450
NO	20 (48.78%)	21 (51.22%)		
YES	105 (55.26%)	85 (44.74%)		
BRAF and TERT mutation			16.244	<0.001
NO	125 (57.34%)	93 (42.66%)		
YES	0 (0.00%)	13 (100.00%)		
RAS and TERT mutation			——	1.000
NO	124 (53.19%)	106 (46.09%)		
YES	1 (100.00%)	0 (0.00%)		
Tumor-Infiltrating Lymphocytes			2.508	0.113
NO	85 (50.90%)	82 (49.10%)		
YES	40 (62.50%)	24 (37.50%)		
Concurrent Hashimoto’s Thyroiditis			3.847	0.0498
NO	105 (51.72%)	98 (48.28%)		
YES	20 (71.43%)	8 (28.57%)		
Age	42.00 (19.00)	41.50 (20.25)	−0.339	0.735
BMI	23.42 (4.71)	23.48 (4.34)	−0.223	0.823
Tumor Size	0.70 (0.50)	1.00 (1.43)	−3.425	<0.001
KI67	2.00 (1.00)	3.00 (1.00)	−1.863	0.062
MLR	0.23 (0.13)	0.24 (0.14)	−0.611	0.541
SIRI	0.85 (0.62)​	​1.15 (0.78)​	−2.668	0.008
SII	500.93 (336.82)	553.32 (477.63)	−1.210	0.226

**TABLE 10:** Multivariate analyses with Lymph node metastasis as the dependent variable.

Variables	B	SE	Wald	P
Gender	0.840	0.329	6.503	0.011
Capsular Invasion	0.192	0.370	0.271	0.603
Multifocality	0.579	0.393	2.170	0.141
Bilaterality	0.468	0.437	1.145	0.285
Lymphovascular Invasion	0.688	0.470	2.146	0.143
Psammoma Bodies	1.144	0.557	4.213	0.040
Non-BRAF mutation	−1.544	0.667	5.353	0.021
BRAF and TERT mutation	20.027	10,395.331	0.000	0.998
Concurrent Hashimoto’s Thyroiditis	−0.714	0.559	1.632	0.201
Tumor Size	0.456	0.230	3.933	0.047
SIRI	0.380	0.150	6.422	0.011

#### 3.3.5 ROC analysis: SIRI predicts lymph node metastasis and SII identifies aggressive pathologic variants

**FIGURE 1:**
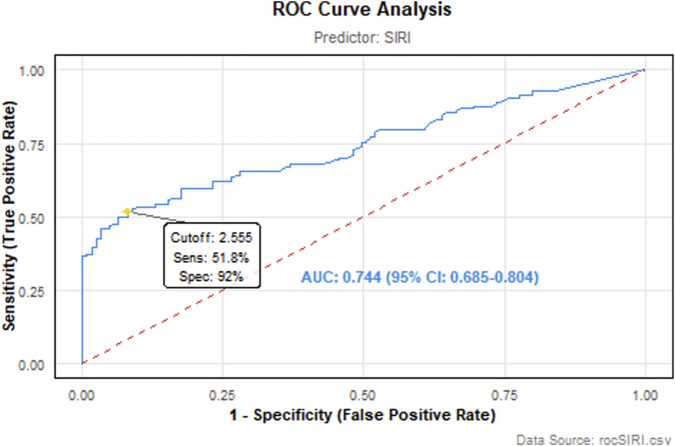
ROC curve analysis of the SIRI for detecting lymph node metastasis in DTC. The AUC was 0.744 (95% CI: 0.685–0.804), indicating moderate accuracy. Using a cutoff of 2.555, SIRI identified metastatic patients with 92.0% specificity and 51.8% sensitivity.

ROC analysis demonstrated that the SIRI exhibited moderate discriminative ability in predicting lymph node metastasis, with an AUC of 0.744 (95% CI: 0.685–0.804). The optimal cutoff value for SIRI was identified as 2.555 through maximization of the Youden index. At this threshold, sensitivity reached 51.8%, while specificity was notably higher at 92.0%, yielding a Youden index of 0.438 (Figure 1).

**FIGURE 2:**
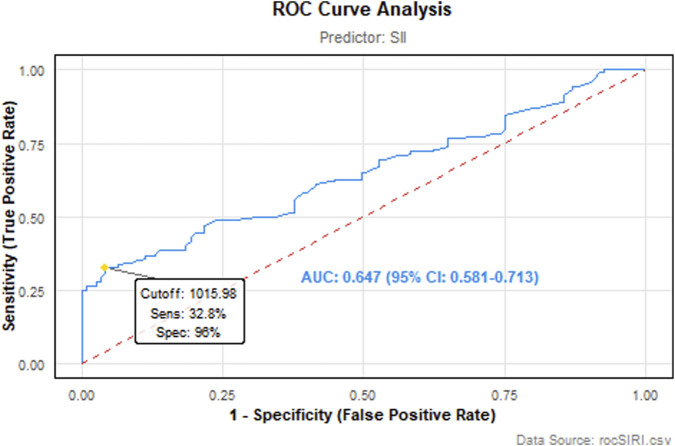
The SII as a specific biomarker for aggressive pathologic variants in DTC. ROC analysis showed an AUC of 0.647 (95% CI: 0.581–0.713). At a cutoff of 1015.98, the SII ruled in aggressive pathologic variants with 96.0% specificity, while sensitivity was 32.8%.

Discriminatory performance of SII ROC analysis revealed that the SII demonstrated moderate discriminatory ability in identifying invasive pathological subtypes, with an AUC of 0.647 (95% CI: 0.581–0.713). The optimal cutoff value was determined as 1,015.98 by maximizing the Youden index. At this threshold, SII achieved exceptionally high specificity of 96.0% (95% CI: 92.4%–98.1%), but low sensitivity of 32.8% (95% CI: 25.3%–41.2%). The resultant Youden index was 0.288, indicating suboptimal overall diagnostic efficacy (Figure 2).

## 4 Discussion

Over the past decade, AS has been established as a novel clinical management strategy for low-risk thyroid cancer. Multiple prospective studies have progressively expanded its applicability and demonstrated favorable outcomes (Ito et al., 2023; Kim et al., 2024; Miyauchi et al., 2023; Miyauchi et al., 2018; Miyauchi and Ito, 2019; Oda et al., 2017; Sasaki et al., 2023). Currently, the clinical significance of various inflammatory indicators, such as the NLR, PLR, and MLR, in the pathogenesis, treatment response, and prognostic evaluation of solid tumors has been extensively investigated (Gao et al., 2024; Hu et al., 2014; Lu et al., 2024; Zhou et al., 2023; Zuo et al., 2023). This study aims to provide a novel perspective on the utility of inflammatory indicators for risk stratification and dynamic monitoring of patients undergoing AS for differentiated thyroid cancer.

The MLR, calculated from absolute peripheral blood monocyte and lymphocyte counts, serves as a hematological biomarker. Peripheral blood monocytes take participate in paracrine signaling to secrete abundant inflammatory cytokines (e.g., TNF-α, IL-1, IL-6) and chemokines. They can be recruited into the tumor tissue thus leading to a decrease in cytotoxic CD8^+^ T cell infiltration. Studies demonstrate that targeted inhibition of monocyte-derived populations within tumors elevates CD8^+^ T-cell density, thereby suppressing growth across multiple solid malignancies (Li et al., 2017; Mitchem et al., 2013). Notably, non-classical monocytes facilitate NK cell recruitment to metastatic sites through secretion of chemokine ligands (CCL3, CCL4, CCL5). The close relationship between monocytes and lymphocytes has led us to increase our focus on MLR. Substantial evidence establishes MLR as an independent prognostic factor across multiple solid malignancies, including lung cancer, urothelial carcinoma, ovarian cancer, and gastrointestinal stromal tumors (GISTs) (Cananzi et al., 2019; Huai et al., 2023; Kovacs et al., 2023; Shao et al., 2020). Our study demonstrates that elevated MLR levels significantly correlate with adverse clinicopathological features in DTC patients. Specifically, patients with high MLR exhibit increased likelihood of psammoma bodies—a histopathological marker strongly associated with LNM and distant spread (Cai et al., 2015; O'Connor et al., 2022). Furthermore, high MLR independently predicts multifocal tumor occurrence (P < 0.05).

The SIRI, calculated from peripheral neutrophil, monocyte, and lymphocyte counts, serves as an indicator of systemic inflammatory status. Elevated SIRI values correlate not only with heightened inflammatory activity but also carry significant biological implications within the tumor microenvironment. Existing research on SIRI has primarily focused on gastric, colorectal, breast and lung cancers, consistently demonstrating a significant association between high SIRI and poorer prognosis in these malignancies (Cao et al., 2023; Ren et al., 2024; Wu and Zhao, 2024; Ye et al., 2024; Zhang and Cheng, 2024). However, investigations into the correlation between SIRI and clinicopathological characteristics specifically in patients with DTC remain limited. Our findings indicate that DTC patients within the high-SIRI group exhibit significantly more adverse clinicopathological characteristics than those in the low-SIRI group. Specifically, patients with high SIRI had larger maximum tumor diameter and a higher prevalence of lymphovascular invasion. The biological underpinning for these associations may involve the roles of inflammatory cells within the tumor microenvironment. Neutrophils and monocytes can secrete various cytokines and enzymes that promote tumor cell proliferation and survival. For instance, inflammatory cytokines like IL-6 can activate the STAT3 signaling pathway, thereby upregulating the expression of anti-apoptotic and proliferative proteins (El-Tanani et al., 2022). Monocytes recruited into tumor tissue differentiate into tumor-associated macrophages (TAMs). TAMs secrete numerous cytokines and matrix metalloproteinases (MMPs), which degrade the extracellular matrix and reduce tumor cell adhesion, thereby facilitating tumor cell invasion and metastasis (Liu et al., 2019). Conversely, lymphocytes, particularly cytotoxic CD8^+^ T cells, are integral to anti-tumor immune responses. A reduction in lymphocyte count impairs immune surveillance, thereby promoting tumor immune evasion and progression (Christo et al., 2024; Galassi et al., 2024; Lawson et al., 2020; Ribot et al., 2021). The ROC analysis reveals that the SIRI exhibits a moderately strong discriminatory capacity (AUC = 0.744; 95% CI: 0.685–0.804) in predicting lymph node metastasis of DTC. Its high specificity (0.92) underscores SIRI’s utility as a reliable tool to rule out non-metastatic patients, which is critical for selecting candidates suitable for active surveillance. However, the suboptimal sensitivity (0.518) implies a substantial risk of false negatives, necessitating caution when using SIRI alone for positive identification. The optimal cutoff value (2.555) derived from the Youden index provides a clinically actionable threshold.

The SII, derived from the combined counts of neutrophils, lymphocytes, and platelets, serves as a metric to assess systemic inflammatory status in patients. Current clinical evidence indicates that elevated circulating neutrophil levels correlate with poor prognosis in cancer patients (Shaul and Fridlender, 2019). There are multiple complex interactions between platelets and tumors, and these interactions play important roles in tumor growth, metastasis, immune escape, and chemotherapy resistance. Platelet-derived TGF-β can induce Epithelial-Mesenchymal Transition (EMT) in tumor cells, thereby enhancing their migratory and invasive potential. Furthermore, platelets promote tumor angiogenesis by releasing pro-angiogenic factors such as VEGF and Angiopoietins (Ang1-2), establishing a microenvironment conducive to tumor growth and dissemination (Filippelli et al., 2022; Gao et al., 2024; Lu et al., 2024; Zhou et al., 2023). Conversely, lymphocytes, particularly CD8^+^ T cells, are pivotal in anti-tumor immunity. They induce tumor cell apoptosis via the Fas/FasL pathway and inhibit metastatic niche formation through the secretion of Granzyme B (Martinez-Lostao et al., 2015). By mediating cytotoxic cell death and suppressing tumor cell proliferation and migration, lymphocytes play a critical role in cancer immunosurveillance and defense (Galassi et al., 2024; Lawson et al., 2020; Ribot et al., 2021). Consequently, the concurrent manifestation of elevated neutrophil counts, increased platelet levels, and reduced lymphocyte counts–indicative of a high SII value–typically signifies a pronounced inflammatory response coupled with a weakened immune response. This pathological interplay facilitates enhanced tumor cell invasion and metastasis, ultimately resulted in poor prognosis for the patient. Our study revealed that among patients younger than 55 years, those in the high-SII group were significantly older than those in the low-SII group. Additionally, the proportion of patients exhibiting aggressive pathologic variants was significantly greater in the high-SII group compared to the low-SII group. The ROC analysis demonstrated that the systemic immune-inflammation index (SII) exhibits a modest discriminatory capacity (AUC = 0.647; 95%CI:0.581–0.713) in distinguishing aggressive pathologic variants of DTC. While the high specificity (0.96) suggests SII effectively excludes non-aggressive cases, its suboptimal sensitivity (0.328) indicates substantial limitations in identifying true aggressive pathologic variants. The Youden index-derived optimal cutoff (1,015.98) yielded a balanced performance metric of 0.288, positioning SII as a potential adjunctive screening tool. However, the considerable false-negative rate implied by low sensitivity warrants caution in standalone application. These findings underscore the need for multimarker validation studies to enhance diagnostic precision for aggressive DTC subtyping.

This study elucidates the pivotal role of SII and SIRI in stratifying DTC patients for active surveillance. The consistently high specificity (SII: 0.96; SIRI: 0.92) of both indices demonstrates robust capability to rule out high-risk phenotypes—a critical prerequisite for identifying candidates suitable for non-operative management. Although suboptimal sensitivity (SII: 0.328; SIRI: 0.518) limits their power to detect all low-risk cases, this characteristic is clinically acceptable in active surveillance paradigms where minimizing false-positive inclusions outweighs the need for exhaustive case identification. The derived optimal cutoffs (SII: 1,015.98; SIRI: 2.55) provide actionable thresholds for implementing these biomarkers in clinical workflows. When integrated with established tools like ultrasonography and molecular testing, SII/SIRI enhance precision in selecting patients with indolent disease biology. Future validation studies should prospectively assess these indices in risk-adapted management protocols, particularly focusing on long-term outcomes of surveillance cohorts stratified by inflammatory profiling.

Limitations of this study include the following: First, the current sample size requires expansion, as the single-center recruitment may introduce selection bias; thus, future multi-center large-sample prospective studies are warranted to validate our findings. Second, the retrospective design entailed subject selection based on pre-existing data rather than random sampling. Third, the favorable prognosis of differentiated thyroid carcinoma (DTC), characterized by rare postoperative recurrence, metastasis, and disease-specific mortality, undermines prediction model reliability—manifested as inflated AUC with wide confidence intervals in ROC analysis and overfitting risks in Cox regression. External validation through multicenter cohorts is imperative.

## 5 Conclusion

In summary, DTC patients with different preoperative MLR levels demonstrated significant differences in the presence of psammoma bodies and multifocal tumors. Patients with different preoperative SIRI levels exhibited significant differences in maximum tumor diameter and the rate of lymphovascular invasion. Furthermore, significant differences in the proportion of aggressive pathologic variants were observed among patients with different preoperative SII levels. These findings suggest that the MLR, SIRI, and SII levels can serve as predictive indicators for clinical-pathological features of DTC. Multivariable analyses confirmed SIRI as an independent predictor of lymph node metastasis and SII as an independent predictor of aggressive pathological variants. Monitoring SIRI and SII may offer new perspectives for optimizing patient selection in thyroid cancer active surveillance.

SII and SIRI demonstrate significant rule-out utility for high-risk DTC phenotypes, establishing their value as triaging tools in active surveillance programs. Despite suboptimal sensitivity, their high negative predictive power supports clinical deployment at derived cutoffs to identify candidates for non-operative management. Integration with imaging and molecular biomarkers is recommended to mitigate false-negative risks. Prospective validation of these indices in risk-stratified surveillance cohorts is warranted.

## Data Availability

The raw data supporting the conclusions of this article will be made available by the authors, without undue reservation.
